# Game theory elucidates how competitive dynamics mediate animal social networks

**DOI:** 10.1186/s12862-024-02302-6

**Published:** 2024-08-30

**Authors:** Frédérique Dubois

**Affiliations:** Université de Montréal, Montréal, Canada

**Keywords:** Animal social network, Competition, Fighting ability assessment, Hawk-dove game, Phenotypic assortment, Predation

## Abstract

**Background:**

While most game theoretical models assume that individuals randomly interact with all other group members, strong evidence indicates that individuals tend to preferentially interact with some of them. The position of an individual in a network affects, among other factors related to survival, its predation risk and competitive success. Here I then modified the Hawk-Dove game to explore the effect of social network structure on competitive strategy of individuals that differ in their fighting ability and may adjust their use of the Hawk, Dove and Assessor tactics to maximize their foraging success when they meet opponents they are connected with.

**Results:**

From randomly generated networks, I demonstrate that phenotypic assortment by fighting ability reduces individuals’ aggressiveness and, as such, favours cooperative interactions. Furthermore, the success of individuals with the weakest fighting ability is usually highest within networks where they most frequently meet opponents with the same fighting ability as their own, suggesting they might benefit from breaking connections with strong contestants. This might be the case when strong contestants systematically rely on the aggressive Hawk tactic or the risk of being predated is low and independent of the number of neighbours. Thus, I extended the model and built a dynamic model to allow individuals not only to adjust their behaviour to local conditions but also to modify the structure of the social network. The number of connections and degree of phenotypic assortment are then affected by ecological factors (e.g. resources value and predation risk), but above all by whether individuals can reliably assess the competitive ability of their opponents and adjust their behaviour accordingly.

**Conclusions:**

These findings provide strong evidence that behaviour can play a key role in shaping network structure and highlight the importance of considering the coevolution of network and behaviour to apprehend its consequences on population dynamics.

**Supplementary Information:**

The online version contains supplementary material available at 10.1186/s12862-024-02302-6.

## Background

Game theory aims to analyse strategic behaviour when an individual’s fitness depends both on the tactic it uses and that of the players it interacts with. As such, it is frequently used to investigate both human and animal behaviour in various contexts [1–4]. A game theoretical approach, for instance, can be applied to intra-specific competition and cooperation, when individuals compete for gaining access to limited resources (e.g. food or territories) that are important for growth, survival and/or reproduction. They must then decide whether they behave aggressively to obtain the whole resources or accept to share them with other contestants. Optimal tactic use in a competitive situation is usually predicted from the Hawk-Dove (HD) game. In its simplest form, the HD game considers dyadic interactions where both players compete for a good (valued at V) and simultaneously decide to play the Hawk or Dove tactics, that consist of behaving aggressively or peacefully, respectively [5]. It predicts that Hawk is a pure Evolutionary Stable Strategy (ESS) when the value of the good exceeds the cost of losing a fight, while both tactics coexist otherwise. Thus, Dove can never be a pure ESS and as such, the HD game fails to predict the frequently observed resource sharing between group members. Since its original formulation, many variants of the HD game have been developed to predict the frequency of agonistic and cooperative interactions under more realistic conditions. For example, it has been modified to consider the fact that resources can be challenged by more than one competitor [6], the same individuals meet repeatedly [7, 8], or are related [9].

Yet, most HD game models assume that individuals are equivalent and interact equally with all other group members, although studies on animal personality and animal social networks have proven none of these hypotheses to be realistic: instead, individuals of the same population most often differ consistently in their behaviour [10, 11] and tend to preferentially interact with certain individuals [12]. Furthermore, several studies have found a link between social network characteristics (e.g. number of connections, degree of phenotypic assortment) and personality traits (e.g. [13–15]), with, for instance, shy individuals having more connections than bold individuals [16, 17]. These findings strongly suggest that the structure of the social network and the behaviour of individuals within the network might interplay with each other, making it thereby necessary to consider the behaviour of individuals to better understand the dynamics of animal social networks.

Specifically, the position of an individual within a social network has consequences on several fitness components and may present both advantages and disadvantages. Furthermore, the relative importance of these advantages and disadvantages is likely to vary according to the phenotypic characteristics of the individual and the ecological conditions it experiences. We would thus expect great variation in social network position (i.e. number of direct connections) among individuals, as well as in group network structure, among ecological contexts [18, 19]. For example, it is well recognized that the risk of an individual to become infected by a disease or a parasite depends on its position in the social network, and particularly, on its contact rate with infected individuals [20, 21]. For that reason, populations with a low disease prevalence should be more connected (i.e. with each individual interacting with a larger number of neighbours) compared with those with a high prevalence, in which individuals would benefit from limiting their interactions with sick individuals [22]. By contrast, being connected to multiple neighbours may reduce the risk of mortality caused by predation not only because highly connected individuals may benefit from a dilution or confusion effect [23, 24] but also because they occupy more central positions within the group and, therefore, have a lower probability of suffering predator attacks compared to less connected and more peripheric individuals [23, 25].

Adjusting the social structure of their network, therefore, is viewed as an adaptive way to cope with changes in predation pressure or disease transmission risk [26, 27]. Yet, considering only ecological factors (e.g. predation pressure or disease prevalence) may be insufficient to predict social network characteristics, as the success of an individual also depends on both its behavioural type (e.g. activity and exploration [28]) and that of its neighbours. For instance, differences among individuals in their social environment may influence their success in competition for resources (e.g. [29, 30]). Notably, when individuals differ in their fighting ability, the chances of gaining access to resources are affected by the fighting ability of the focal individual and that of its neighbours, as well as the competitive tactic they use. The abundance and richness of food resources, therefore, might play a key role in shaping social network structure, as predation pressure does. For instance, ecological conditions that favour aggressive behaviour (e.g. high-quality resources) might favour groups where individuals mainly interact with neighbours with the same phenotype as their own (high degree of phenotypic assortment), which in turn should reduce the benefits of being aggressive.

Here I then first modified the HD game to explore the effect of social network structure on competitive tactic use and average success, by assuming that individuals’ probability of survival (used as a proxy of fitness) depends on their ability to both gain food and escape predators. Specifically, I introduced variation in fighting ability and randomly generated social networks in which individuals can only meet conspecifics they are directly connected with. I assumed that when two opponents play Hawk, their chance of winning the fight depends on their respective fighting abilities. To allow individuals to avoid a fight that they would have no chance of winning, I then introduced, as an alternative tactic choice, the Assessor tactic, which consists in gauging the fighting ability of their opponent before deciding to behave aggressively or peacefully. I demonstrate that individuals’ optimal tactic use, as well as their expected success, depend on both ecological (i.e. quantity of energy that can be gained from a resource or cost of fighting) and social (i.e. degree of phenotypic assortment) factors. These findings support the idea that some individuals should not only adjust their behaviour to local conditions but also modify the structure of the social network. I then extended the game and built a dynamic model to predict the combined effects of competition and predation on social network structures and frequency of use of aggressive behaviours.

## The model

### A. Predicting optimal competitive tactic use

The model simulates a group of individuals embedded within a social network in which each player only competes with the members of the group with whom it is connected. Individuals are characterized by their fighting ability (i.e. high, intermediate or low) and their competitive strategy (i.e. the frequency with which they use the Hawk, Dove and Assessor tactics). All individuals of a given phenotype have the same fighting ability and use the same competitive strategy. While the fighting ability of individuals remains constant over time, the strategy adopted by the individuals of each phenotype can evolve over time until an equilibrium is reached where the strategy is optimal for each phenotype within the considered social network. To find the optimal competitive strategies, I therefore calculate the average quantity of resources gained by individuals of each phenotype (as a measure of fitness) and determine, in turn for each phenotype, the optimal frequencies of use of each tactic. The same analysis is repeated, for each set of parameter values, on 100 randomly simulated social networks.

Specifically, I consider a group of $3n$ individuals (all parameters are listed in Table 1) embedded within a social network: individuals differ in their fighting ability $f$, which is modelled as a discrete variable taking three values (i.e. $f$ =1, 2 or 3) for individuals with a high, intermediate or low fighting ability, and $n$ represents the number of individuals of each phenotype. Individuals compete in pairs with individuals with whom they are connected, for gaining access to resources whose value is denoted $V$. The structure of a social network, randomly generated in each simulation, is described by its adjacency matrix $M$:

**Table 1:** Definition of the parameters and state variables used in the model. For all parameters, the default value or range of tested values is specified

Symbol	Meaning
$n$	Number of individuals of each phenotype (default value: 6)
$V$	Value of the contested resources (range of tested values: 2–20)
$c$	Cost of fighting (range of tested values: 1–15)
$a$	Cost of assessment (range of tested values: 0–10)
$\delta$	Risk of being detected by a predator (range of tested values: 0–1)
$d$	Confusion factor (range of tested values: 0–1)
*f*	Fighting ability of individual $i$ (1-high, 2-intermediate, 3-low)
${v}_{i,j}$	Link between players $i$ and $j$ (0-disconnected, 1-connected)
${k}_{i}$	Degree of individual $i$ (i.e. number of connexions)
${m}_{f}$	Average risk of mortality of individuals whose fighting ability is $f$
${k}_{i1}$	Number of connexions of individual $i$ with strong contestants
${k}_{i2}$	Number of connexions of individual $i$ with intermediate contestants
${k}_{i3}$	Number of connexions of individual $i$ with weak contestants
${\overline{k} }_{f}$	Average number of connexions of individuals whose fighting is $f$
${\overline{k} }_{f1}$	Average number of connexions of individuals whose fighting is $f$ with strong contestants
${\overline{k} }_{f2}$	Average number of connexions of individuals whose fighting is $f$ with intermediate contestants
${\overline{k} }_{f3}$	Average number of connexions of individuals whose fighting is $f$ with weak contestants
${x}_{f}$	Frequency with which individuals of fighting ability $f$ use the Hawk tactic
${y}_{f}$	Frequency with which individuals of fighting ability $f$ use the Dove tactic
${z}_{f}$	Frequency with which individuals of fighting ability $f$ use the Assessor tactic
${W}_{f}$	Average fitness of individuals of fighting ability $f$
$\sigma$	Proportion of resources shared without aggression

$$M=\left[\begin{array}{ccc}{v}_{11}& \dots & {v}_{1n}\\ \dots & \dots & \dots \\ {v}_{n1}& \dots & {v}_{nn}\end{array}\right]$$

Each element of the matrix ${v}_{ij}$ can take the value of 1 or 0 if the two individuals (i.e. $i$ and $j$) are connected or not, respectively. To randomly generate a social network, I therefore randomly draw a value (i.e. 0 or 1) for each pair of individuals, with each possible connection between players being realized with probability 0.5.

The degree of each individual $i$ (i.e. the number of connections it has with other individuals, ${k}_{i}$), can then be obtained directly from the elements of the adjacency matrix as the sum of either the rows or the columns of the matrix:

$${k}_{i}=\sum_{j=1}^{3n}{v}_{ij}=\sum_{i=1}^{3n}{v}_{ji}$$

Among all the connections of an individual, one can distinguish those maintained with neighbours with a high (i.e. ${k}_{i1}$), intermediate (i.e. ${k}_{i2}$) and low (i.e. ${k}_{i3}$) fighting ability with: ${k}_{i}={k}_{i1}+{k}_{i2}+{k}_{i3}$. For each player whose fighting ability is $f$, one can also determine its degree of phenotypic assortment (i.e. the proportion of connections with similar contestants) which equals: ${}^{{k}_{if}}\!\left/ \!{}_{{k}_{i}}\right.$.

The average values per fighting ability are then calculated and denoted by the variables: ${\overline{k} }_{f}$, ${\overline{k} }_{f1}$, ${\overline{k} }_{f2}$ and ${\overline{k} }_{f3}$.

Individuals that are connected within the social network interact by pairwise interactions using the Hawk, Dove or Assessor tactics to try to appropriate all or part of the contested resource. The model assumes that an individual playing Dove behaves peacefully and so share the resource with another Dove player but retreats if it interacts with an aggressive Hawk player. By contrast, if two Hawk players interact with each other, they engage in a fight that incurs a metabolic cost $c$ to both players [31], but at the end of which one player gets the entire resource. When the two players differ in their fighting ability, the contestant with the strongest ability always wins the fight (see payoff matrix in Table 2), while the two contestants have a 50% chance of winning the resource when they have the same ability (see payoff matrix in Table 3). Finally, Assessor players first assess the fighting ability of their opponent before deciding to behave aggressively or peacefully. During the time devoted to assessment, the contestant being assessed can begin to exploit the resource if it adopts a fixed tactic, and thus obtains a quantity $a$ (i.e. the cost of assessment) of the resource, before the assessor decides how to behave. Yet, if both contestants adopt the Assessor tactic, they do not suffer the cost of assessment since neither player can start exploiting the resource before the other contestant. If the two players differ in their fighting ability, once the assessment is over, an Assessor player decides to behave aggressively if its fighting ability is stronger than that of its opponent, but peacefully otherwise, in which case it withdraws (see payoff matrix in Table 2). By contrast, if the two players have the same fighting ability, an Assessor player plays Hawk or Dove with the same probability (see payoff matrix in Table 3).

**Table 2:** Payoff matrix for two players that differ in their fighting ability. For each interaction, the first line represents the average payoff of the player with the weakest fighting ability (i.e. ${E}_{w}$), while the second line corresponds to average payoff of the player with the strongest fighting ability (i.e. ${E}_{s}$)

	Hawk	Dove	Assessor
Hawk	${E}_{w}\left(H,H\right)=-c$ ${E}_{s}\left(H,H\right)=V-c$	${E}_{w}\left(H,D\right)=V$ ${E}_{s}\left(D,H\right)=0$	${E}_{w}\left(H,A\right)=a-c$ ${E}_{s}\left(A,H\right)=V-a-c$
Dove	${E}_{w}\left(D,H\right)=0$ ${E}_{s}\left(H,D\right)=V$	${E}_{w}\left(D,D\right)=\frac{V}{2}$ ${E}_{s}\left(D,D\right)=\frac{V}{2}$	${E}_{w}\left(D,A\right)=a$ ${E}_{s}\left(A,D\right)=V-a$
Assessor	${E}_{w}\left(A,H\right)=0$ ${E}_{s}\left(H,A\right)=V$	${E}_{w}\left(A,D\right)=\frac{V-a}{2}$ ${E}_{s}\left(D,A\right)=\frac{V+a}{2}$	${E}_{w}\left(A,A\right)=0$ ${E}_{s}\left(A,A\right)=V$

**Table 3:** Payoff matrix for two players with the same fighting ability (i.e. equal competitors). The expressions in each cell of the matrix represent the average payoffs of the row player

	Hawk	Dove	Assessor
Hawk	${E}_{e}\left(H,H\right)=\frac{V}{2}-c$	${E}_{e}\left(H,D\right)=V$	${E}_{e}\left(H,A\right)=\frac{3V+a-c}{2}$
Dove	${E}_{e}\left(D,H\right)=0$	${E}_{e}\left(D,D\right)=\frac{V}{2}$	${E}_{e}\left(D,A\right)=\frac{V+3a}{4}$
Assessor	${E}_{e}\left(A,H\right)=\frac{V-a}{4}-\frac{c}{2}$	${E}_{e}\left(A,D\right)=\frac{3V-3a}{4}$	${E}_{e}\left(A,A\right)=\frac{2V-c}{4}$

The frequency at which the players with fighting ability $f$ use each competitive tactic, denoted as $\left\{{x}_{f},{y}_{f},{z}_{f}\right\}$ with: ${x}_{f}+{y}_{f}+{z}_{f}=1$, affects their average success (i.e. ${W}_{f}$) that can be calculated from expressions in Tables 2 and 3 as:

1 $${W}_{f}=\left\{{x}_{f}\times {W}_{f}\left(H\right)+{y}_{f}\times {W}_{f}\left(D\right)+{z}_{f}\times {W}_{f}\left(A\right)\right\}\times \left(1-{m}_{f}\right)$$

In the above equation, the last term outside braces corresponds to the average probability of individuals whose fighting ability is $f$ to escape predation (see explanations below), while the first, second and third terms into braces represent their average foraging success when playing Hawk (i.e. ${W}_{f}(H)$), Dove (i.e. ${W}_{f}(D)$) and Assessor (i.e. ${W}_{f}(A)$), with probabilities ${x}_{f},$ ${y}_{f}$ and ${z}_{f}$, respectively. These values depend on both the foraging tactic and the fighting ability of their opponent. For instance, the average foraging success of a weak individual (i.e. with $f=3$) that plays Hawk can be estimated as:

2 $$\begin{array}{c}{W}_{3}\left(H\right)=\frac{{\overline{k} }_{33}}{{\overline{k} }_{3}}\times \left\{{x}_{3}\times {E}_{e}\left(H,H\right)+{y}_{3}\times {E}_{e}\left(H,D\right)+{z}_{3}\times {E}_{e}\left(H,A\right)\right\}\\ +\frac{{\overline{k} }_{32}}{{\overline{k} }_{3}}\times \left\{{x}_{2}\times {E}_{w}\left(H,H\right)+{y}_{2}\times {E}_{w}\left(H,D\right)+{z}_{2}\times {E}_{w}\left(H,A\right)\right\}\\ +\frac{{\overline{k} }_{31}}{{\overline{k} }_{3}}\times \left\{{x}_{1}\times {E}_{w}\left(H,H\right)+{y}_{1}\times {E}_{w}\left(H,D\right)+{z}_{1}\times {E}_{w}\left(H,A\right)\right\}\end{array}$$

Specifically, the first line of Eq. (2) corresponds to the average foraging success of a weak contestant when interacting with another weak contestant, and so when both players have the same fighting ability (Table 3), while the two other lines correspond to its average foraging success when meeting an individual whose fighting ability is intermediate or high and has, in both cases, a weaker ability than its opponent (Table 2).

The average success of individuals with fighting ability $f$, as shown in Eq. (1), is reduced by their average risk of mortality due to predation (i.e. ${m}_{f}$). More precisely, all players, irrespective of their fighting ability, have a probability $\delta$ of being detected by a predator. Furthermore, the chances of escaping a predator are influenced by the presence of neighbours (but not their phenotype) and to what extent their presence affects the predator’s success rate (i.e. the confusion factor $d$, with $d\ge 0$). Thus, the average mortality rate of individuals with fighting ability $f$ that are connected on average with ${\overline{k} }_{f}$ neighbours can be estimated as:

$${m}_{f}=\frac{\delta }{{{\overline{k} }_{f}}^{d}}$$

According to this equation, the rate of success of a predator is independent of the number of players connected when $d$ is equal to zero, but decreases as $d$ increases, through early predator detection and confusion effects.

To predict the optimal frequencies of use of the three competitive tactics for individuals of each fighting ability (i.e. $\left\{{x}_{f}^{*},{y}_{f}^{*},{z}_{f}^{*}\right\}$), I first fix the strategy of the players whose fighting abilities are intermediate and strong (i.e. $\left\{{x}_{2},{y}_{2},{z}_{2}\right\}$ and $\left\{{x}_{1},{y}_{1},{z}_{1}\right\}$), and I calculate the average success of weak competitors for all the combinations of values ​​of $\left\{{x}_{3},{y}_{3},{z}_{3}\right\}$. I then retain that maximizing ${W}_{3}$. Then, knowing $\left\{{x}_{3}^{*},{y}_{3}^{*},{z}_{3}^{*}\right\}$ (and $\left\{{x}_{1},{y}_{1},{z}_{1}\right\}$), I seek the best response for individuals with an intermediate fighting ability. To do that, I calculate, as above, their average success for all the combinations of values ​​of $\left\{{x}_{2},{y}_{2},{z}_{2}\right\}$ and I retain that maximizing ${W}_{2}$. Then, knowing $\left\{{x}_{2}^{*},{y}_{2}^{*},{z}_{2}^{*}\right\}$ and $\left\{{x}_{3}^{*},{y}_{3}^{*},{z}_{3}^{*}\right\}$, I seek the best response for individuals with a high fighting ability by calculating their average success for all the combinations of values ​​of $\left\{{x}_{1},{y}_{1},{z}_{1}\right\}$ and then retaining that maximizing ${W}_{1}$. I repeat the same procedure until finding the equilibrium combinations of values of $\left\{{x}_{1}^{*},{y}_{1}^{*},{z}_{1}^{*}\right\}$, $\left\{{x}_{2}^{*},{y}_{2}^{*},{z}_{2}^{*}\right\}$ and $\left\{{x}_{3}^{*},{y}_{3}^{*},{z}_{3}^{*}\right\}$. Note that the equilibrium is always achieved and that the equilibrium values ​​are independent of the order in which the three phenotypes are considered. From the optimal strategies for each phenotype, I then deduce the proportion of resources that should be shared without aggression, as:

3 $$\sigma =\frac{{\overline{k} }_{11}}{{\overline{k} }_{1}}\times \left({{y}_{1}}^{2}+\frac{{{z}_{1}}^{2}}{4}\right)+\frac{{\overline{k} }_{22}}{{\overline{k} }_{2}}\times \left({{y}_{2}}^{2}+\frac{{{z}_{2}}^{2}}{4}\right)+\frac{{\overline{k} }_{33}}{{\overline{k} }_{3}}\times \left({{y}_{3}}^{2}+\frac{{{z}_{3}}^{2}}{4}\right)+\left({y}_{1}{y}_{2}\right)\times \left(\frac{{\overline{k} }_{12}}{{\overline{k} }_{1}}+\frac{{\overline{k} }_{21}}{{\overline{k} }_{2}}\right)+\left({y}_{1}{y}_{3}\right)\times \left(\frac{{\overline{k} }_{13}}{{\overline{k} }_{1}}+\frac{{\overline{k} }_{31}}{{\overline{k} }_{3}}\right)+\left({y}_{2}{y}_{3}\right)\times \left(\frac{{\overline{k} }_{23}}{{\overline{k} }_{2}}+\frac{{\overline{k} }_{32}}{{\overline{k} }_{3}}\right)$$

The first three products correspond to the interactions between equal competitors, that can share the resource if either they both play Dove (i.e. with probability ${{y}_{f}}^{2}$) or they both play Assessor and decide to behave non-aggressively after having assessed the fighting ability of their opponent (i.e. with a probability of ${z}_{f}/2$ for each contestant).The last three products correspond to the interactions between contestants with different fighting abilities, that can share the resource only if they both play Dove.

I ran the model by varying the different parameters to explore their effects on the optimal competitive strategy and fitness of each phenotype and the proportion of peacefully shared resources. For each set of parameter values, I generated 100 random networks, and then I calculated the averaged values from the 100 simulations. Given that the 100 randomly generated social networks differ among each other in terms of average degree of assortment for each phenotype, I also explored the influence of this factor on the average frequency of use of the 3 competitive tactics and the average success of each phenotype, based on the results of the 100 simulations obtained for a given set of parameter values.

### B. Examining the evolution of network structure

In the previous model, I considered a fixed randomly generated network structure and allowed individuals to adjust their competitive tactic use to their social environment. Now, I also assume that individuals can modify the structure of their social network, by forming new connections or breaking existing ones. To do that, I first determine the optimal strategy for each phenotype in the initial social network in which all individuals are connected to each other, using the same procedure as above, and I deduce their average respective success. Then, at each subsequent time step, I randomly choose two individuals ($i$,$j$) that I either disconnect or reconnect (if ${v}_{ij}$=1 or 0, respectively). For this novel social network (in which the randomly chosen connection is broken or formed), I seek again the optimal strategy for each phenotype, and I deduce their average respective success. To decide whether the broken (or formed) connection is re-established (or re-broken), I then compare the average success of the phenotype of the chosen individual in the novel network to its average success in the previous network and keep the network with the highest average success. The same procedure is repeated for 100 connections, randomly chosen at each time. I then obtain the final network for which I calculate the average number of neighbours for each phenotype as well as its degree of phenotypic assortment. I varied the cost of assessment ($a=1 \text{or} 10$), the cost of fighting ($c=2, 8 \text{or} 15$), the probability of being detected by a predator ($\delta =0.2 \text{or} 0.8$) as well as the factor of confusion ($d=0.2 \text{or} 1$), for a total of 24 combinations of parameter values, and run 20 simulations for each combination.

## Results

### A. Predicting optimal competitive tactic use

**Fig. 1:**
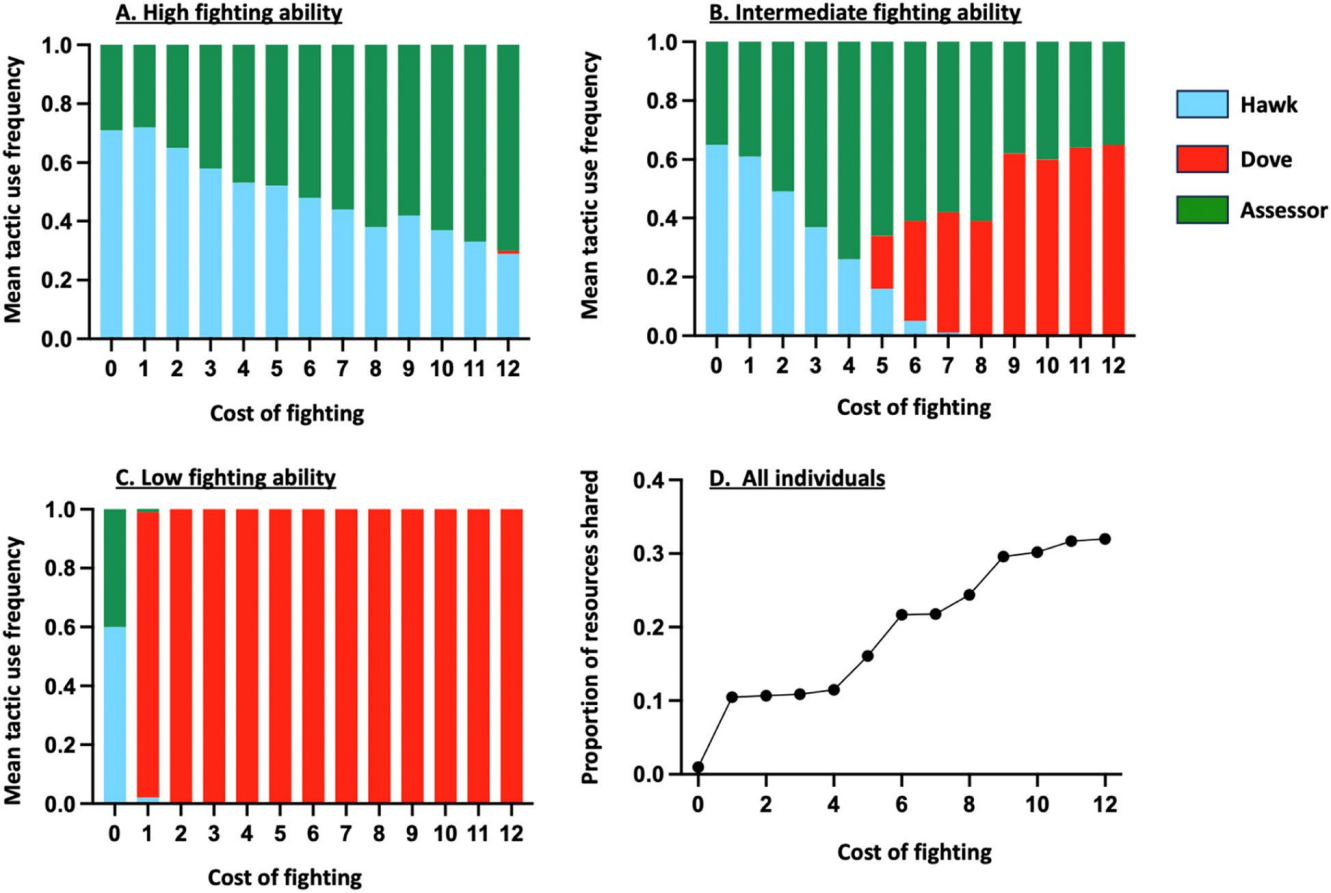
Effects of the cost of fighting (i.e. $c$) on optimal competitive strategy for individuals with a high (**A**), intermediate (**B**) and low (**C**) fighting ability, and on the proportion of resources shared without aggression (**D**). In the figure: $n=6$, $V=10$ and $a=2$. The reported values represent the average frequencies of use of the three tactics estimated from 100 fixed randomly generated social networks for each set of parameter values

The model assumes that predation reduces the success of individuals but without causing deaths. For this reason, predation parameters (i.e. $\delta$ and $d$) have no impact on the optimal competitive strategies, contrary to the parameters which affect the success of individuals during dyadic interactions. Because a fight induces an energetic cost, regardless of its outcome, the model predicts notably that an animal should behave peacefully, and as such avoid fights, when its chances of winning a fight are low and the amount of energy it can get from a resource is insufficient to cover the cost of fighting. For that reason, the frequency of use of the Dove tactic is higher among individuals with a low fighting ability (Fig. 1C), compared to those with an intermediate (Fig. 1B) or high (Fig. 1A) fighting ability. In addition, increasing the cost of fighting leads to an increase in the mean Dove tactic use and, hence, in the proportion of resources that are shared without aggression (Fig. 1D). Conversely, increasing V (the value of the contested resources) leads to a decrease in the proportion of peacefully shared resources (Fig. S1 and Fig. S2). This effect is particularly pronounced when individuals may rely on the Assessor tactic (i.e. low cost of assessment, Fig. S1) compared to when they cannot (i.e. high cost of assessment, Fig. S2). When the cost of assessment is low, indeed, individuals whose fighting ability is intermediate and high mainly rely on the Assessor tactic when they compete for resources of great value (Fig. S1). Resources, consequently, should never be shared between asymmetric contestants. When the cost of assessment is larger (Fig. S2), individuals cannot rely anymore on the Assessor tactic. Individuals whose fighting ability is low and intermediate then mainly use the peaceful Dove tactic, regardless of the value of the contested resources, while strong contestants increase their use of the Hawk tactic as V increases. Thus, increasing resource value also leads to a reduction in the proportion of shared resources, though to a lesser extent, as food sharing then occurs when the two opponents have both a low or an intermediate fighting ability (Fig. S2).

**Fig. 2:**
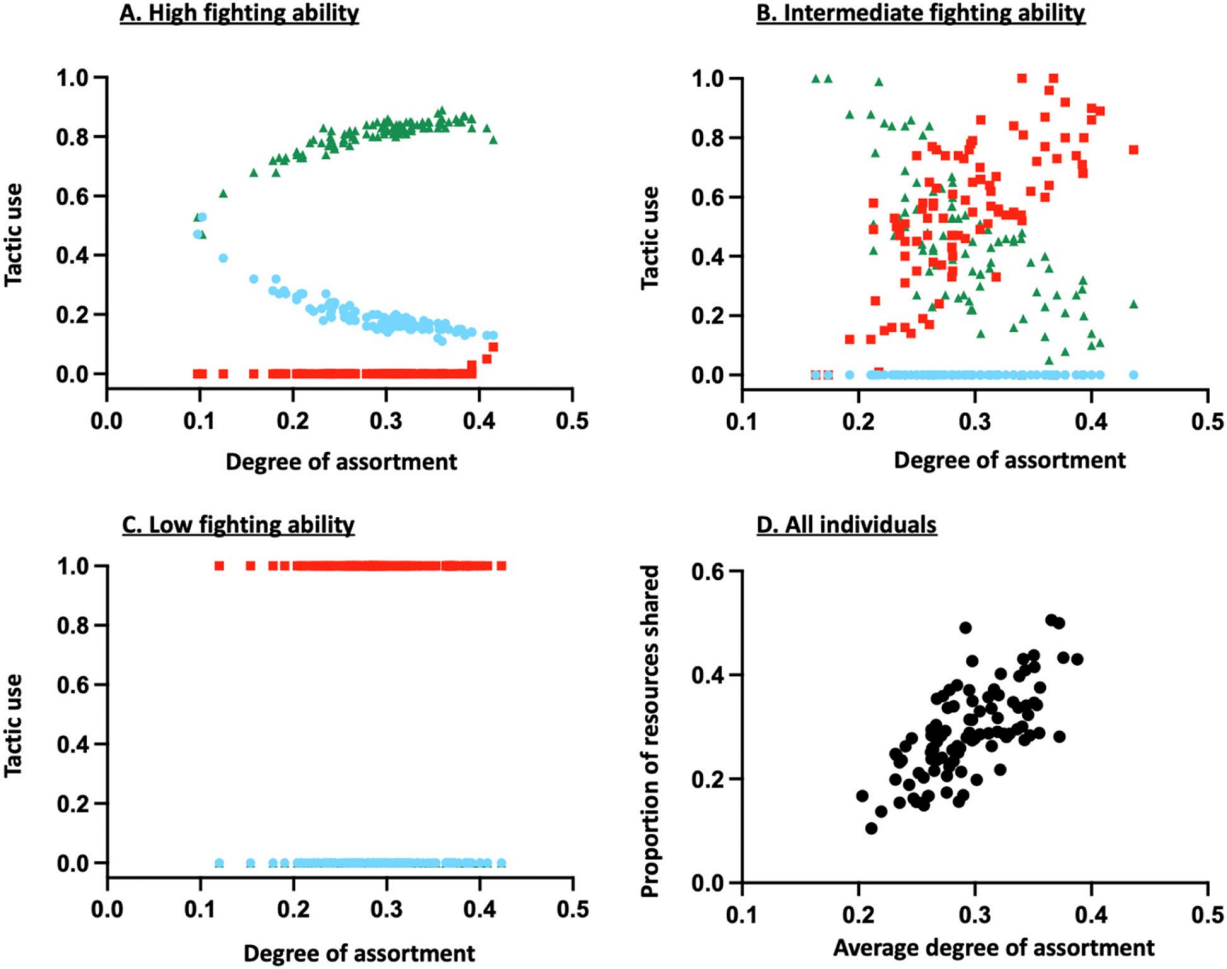
Effects of the degree of phenotypic assortment (i.e. proportion of connections with similar contestants ${k}_{if}/{k}_{i}$) of individuals with a high (**A**), intermediate (**B**) and low (**C**) fighting ability on their optimal competitive strategy and influence on the mean degree of phenotypic assortment on the proportion of resources shared without aggression (**D**). The blue, red and green symbols represent, respectively, the Hawk, Dove and Assessor tactics. The results were obtained from 100 fixed randomly generated social networks with the same parameter values that is: $n=6$, $V=8$, $c=2$ and $a=1$

**Fig. 3:**
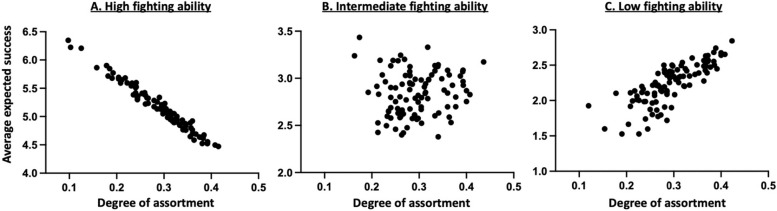
Effect of the degree of phenotypic assortment (i.e. proportion of connections with similar contestants ${k}_{if}/{k}_{i}$) of individuals with a high (**A**), intermediate (**B**) and low (**C**) fighting ability on their average success. The results were obtained from 100 fixed randomly generated social networks with the same parameter values that is: $n=6$, $V=8$, $c=2$, $a=1$, $\delta =0.5$ and $d$=1

Furthermore, whatever the resource value, the cost of a fight or the cost of assessment, the model predicts that the proportion of shared resources should increase as the average degree of assortment increases (Fig. 2D). This arises because individuals are expected to increasingly rely on the Dove or Assessor tactic as their probability of meeting opponents with the same fighting ability increases (Fig. 2). For that reason, the model predicts that the average success of individuals should be affected by the structure of the social network, and especially by the degree of phenotypic assortment of individuals, though differently depending on their phenotype. Indeed, increasing the degree of phenotypic assortment among strong contestants leads to a decrease in their average success (Fig. 3). This may be explained by the fact that individuals with the strongest fighting ability win the contested resource when they meet a weaker opponent, and for that reason achieve their highest success when they most often interact with opponents with a different phenotype as their own. Conversely, individuals with the weakest fighting ability mainly rely on the Dove tactic and thus achieve their highest success when they interact most often with opponents with the same ability, with whom they can share the resources (Fig. 3). Thus, under most conditions, one might expect strong competitors to have very few neighbours and a low degree of phenotypic assortment, compared to weak contestants.

### B. Examining the evolution of network structure

**Fig. 4:**
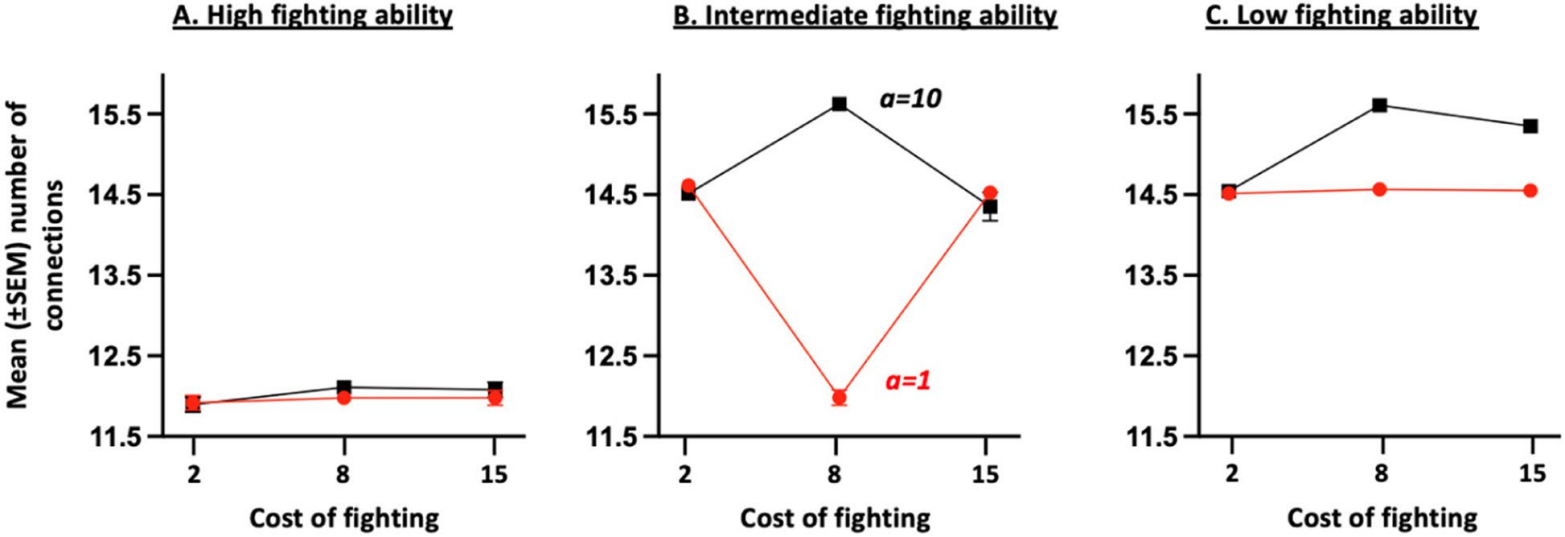
Mean ($\pm \text{SEM}$) number of connections in the final social network for individuals with a high (**A**), intermediate (**B**) and low (**C**) fighting ability in relation to the cost of fighting (i.e. $c$) and the cost of assessment (i.e. $a$). The final network is obtained after having allowed 100 times in a row, a randomly chosen individual to disconnect from (or reconnect with) one of its neighbors, also randomly chosen. The averages and standard deviations ​​were calculated from the results obtained for the 24 combinations of parameter values; each combination having been run 20 times. For all combinations of parameter values: $n=6$ and $V=10$

**Fig. 5:**
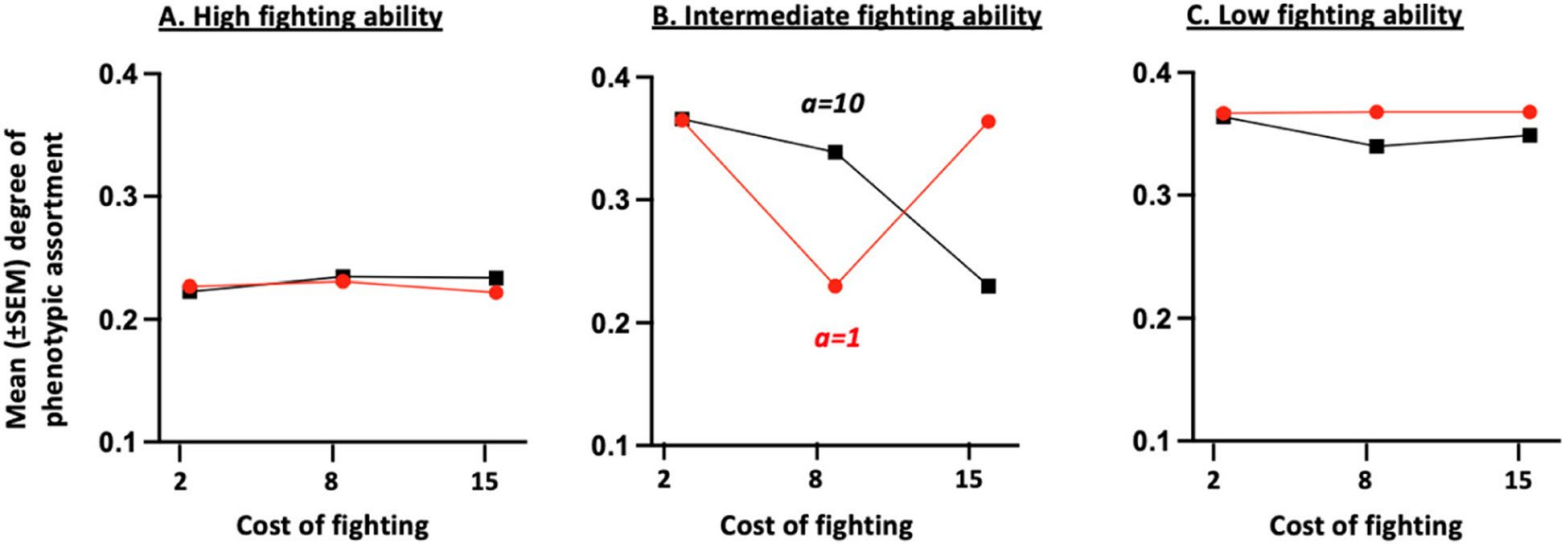
Mean ($\pm \text{SEM}$) degree of phenotypic assortment for individuals with a high (**A**), intermediate (**B**) and low (**C**) fighting ability in the final social network, in relation to the cost of fighting (i.e. *c*) and the cost of assessment (i.e. $a$). The final network is obtained after having allowed 100 times in a row, a randomly chosen individual to disconnect (or reconnect) from one of its neighbors, also randomly chosen. The averages and standard deviations ​​were calculated from the results obtained for the 24 combinations of parameter values; each combination having been run 20 times. For all combinations of parameter values: $n=6$ and $V=10$

When individuals may form and break connections to maximize their average success, the network, predictably, always contains fewer connections for individuals with a high fighting ability (Fig. 4A) compared to those with an intermediate (Fig. 4B) or low (Fig. 4C) ability. Individuals with a low fighting ability also have a higher degree of phenotypic assortment (Fig. 5C) compared to those with a high (Fig. 5A) and, to a lesser extent, intermediate (Fig. 5B) fighting ability. This arises because individuals (including those with a high fighting ability) do benefit, in many conditions, from limiting their interactions with the strongest competitors that, consequently, tend to become more disconnected from others. Yet, the structure of the social network (i.e. the average degree of phenotypic assortment and number of connections of each phenotype) changes according to conditions, essentially depending upon the cost of assessment (and hence, on whether individuals may use the Assessor tactic), and the cost of fighting.

More precisely, when the cost of assessment is high, the number of connections in the final social network is much higher for individuals with low and intermediate fighting abilities (Fig. 4B, C) compared to those with high ability (Fig. 4A). The difference is particularly pronounced for intermediate fighting cost values. A high assessment cost prevents individuals from using the Assessor tactic (Fig. S2). Individuals, instead, rely on the Hawk and Dove tactics, though at different frequencies depending on their phenotype and the cost of fighting. Specifically, individuals with a high fighting ability mainly rely on the aggressive Hawk tactic, making it beneficial for individuals with low and intermediate fighting ability to disconnect from them. Individuals with an intermediate fighting ability also mainly rely on the Hawk tactic when the cost of fighting is low but increase their use of the Dove tactic when the cost of fighting increases, thus promoting the maintenance of connections between individuals with intermediate or low fighting ability. When the cost of fighting increases further until exceeding the value of winning, however, the number of connections and degree of phenotypic assortment tend to decrease for individuals of intermediate individuals (Fig. 4B). This arises because intermediate individuals continue to use the Hawk tactic, that gives them an advantage when competing with weaker contestants. Yet, being aggressive can be very costly when meeting another player of the same competitive ability. For that reason, an increase in the cost of fighting leads to a reduction in the degree of assortment of intermediate individuals as well as a reduction in their number of connections within the social network.

**Fig. 6:**
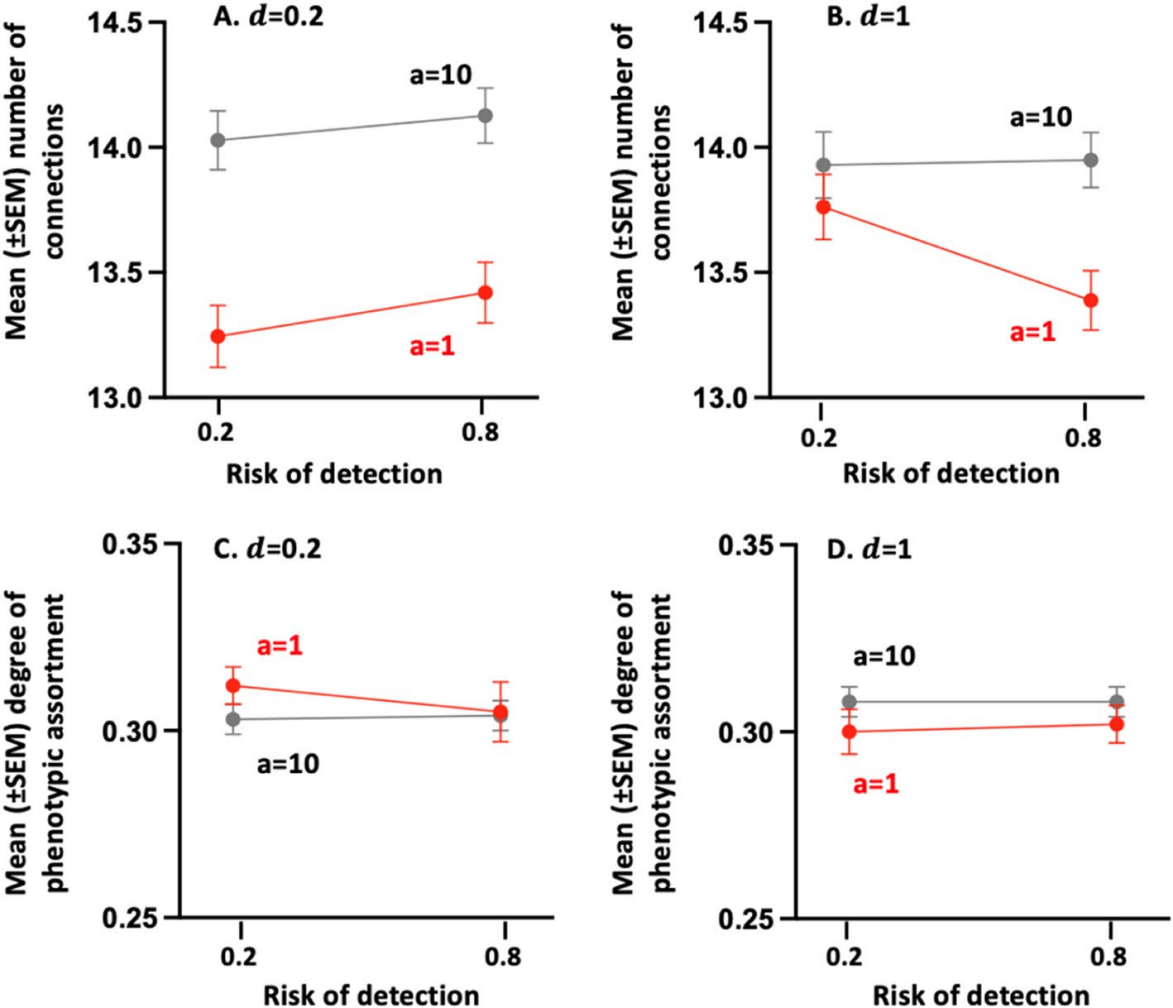
Mean ($\pm \text{SEM}$) number of connection (Panels **A**-**B**) and mean ($\pm \text{SEM}$) degree of phenotypic assortment (Panels **C**-**D**) in the final social network, in relation to the risk of being detected by a predator (i.e. $\sigma$), the confusion effect (i.e. $d$) and cost of assessment (i.e. $a$). The final network is obtained after having allowed 100 times in a row, a randomly chosen individual to disconnect (or reconnect) from one of its neighbors, also randomly chosen. The averages and standard deviations ​​were calculated from the results obtained for the 24 combinations of parameter values; each combination having been run 20 times. For all combinations of parameter values: $n=6$ and $V=10$

By contrast, when the cost of assessment is low, the number of connections (Fig. 5B) and degree of phenotypic assortment (Fig. 6B) of individuals with intermediate fighting ability are at their lowest for intermediate fighting cost values, that is when their frequency of use of the Assessor tactic is highest. Individuals whose fighting ability is high and intermediate, indeed, mainly rely on the Hawk tactic when the cost of fighting is small (Fig. 1A, B). Consequently, individuals with low and intermediate fighting ability then benefit from breaking connections with stronger neighbours and thus tend to have a high degree of phenotypic assortment. When the cost of fighting increases until exceeding the value of winning, individuals must avoid fighting by relying more on the Assessor tactic. Assessor players, however, benefit from avoiding interacting with equal contestants, since an Assessor, by definition, plays Hawk or Dove with the same probability when it encounters an opponent with the same fighting ability as its own. Two Assessor players with the same ability might thus engage in a costly fight. For that reason, an increase in the cost of fighting leads to a reduction in the degree of assortment of intermediate individuals as well as a reduction in their number of connections within the social network. When the cost of fighting increases again, intermediate individuals mainly rely on the Dove tactic, and hence remain connected with individuals whose fighting capacity is equal to or lower than their own.

Finally, the average number of connections is expected to increase with the probability of being detected by a predator, because individuals do benefit from staying connected with more neighbours to reduce their mortality rate caused by predation. This is particularly so when the confusion effect is weak (Fig. 6A), forcing individuals to interact with many other group members to increase their efficiency to escape predators, once detected. Yet, predation has little or no effect on the social network structure in most conditions. This is because predation pressure does not affect the optimal competitive strategy of individuals. Thus, it is only when connections between the different phenotypes are maintained (e.g. when the cost of assessment prevents individuals from adjusting their tactic use according to their opponent’s phenotype) that predation may contribute to maintaining even more connections, but without noticeable effect on the degree of phenotypic assortment (Fig. 6C, D).

## Discussion

The structure of the social network and the behaviour of individuals within the network interplay with each other, as social network structure is a determinant of behaviour, but behaviour in turn plays a key role in shaping network structure. Although based on simplifying assumptions, the present study highlights the importance of considering the coevolution of network and behaviour to make more accurate predictions and apprehend its consequences on population dynamics.

Results from the static model have demonstrated that the social organization of groups affects the use of aggressive behaviour and, as such, influences the frequency of agonistic and cooperative interactions. Indeed, when individuals have a low degree of phenotypic assortment, and hence interact with all group members, the frequency of cooperative interactions is expected to be very low, notably if either the cost of fighting is low, but the cost of assessment is high or conversely if the cost of fighting is high, but the cost of assessment is low. In the first case (i.e. low value of $c$, but high value of $a$), individuals with an intermediate and high fighting ability should mainly use the Hawk aggressive tactic, so that food resources are shared only when two individuals with a weak fighting ability meet. In the second case (i.e. high value of $c$, but low value of $a$), although the cost of fighting is high, individuals with an intermediate and high fighting ability may use the alternative Assessor tactic and, therefore, are expected to behave aggressively when they meet an opponent with a weaker fighting ability as their own. For that reason, only interactions between equal competitors, whatever their fighting ability, may be cooperative. Thus, in every condition, we predict that the proportion of cooperative interactions, in which both players share the resources without overt aggression, should increase as the degree of phenotypic assortment increases.

Assortative interactions have already been proposed as a possible mechanism for the evolution of cooperation by Wilson & Dugatkin [32], but for a different reason. Indeed, in Wilson & Dugatkin’s model [32], individuals differ in their tendency to cooperate, which is thus considered as an intrinsic individual trait, and are free to choose the individuals they interact with. If individuals know the phenotype of the other group members, Wilson & Dugatkin’s model [32] predicts that cooperators should interact with each other by choice, thus forcing cheaters to interact with each other by default. By contrast, in the present study, the propensity to cooperate is not a fixed individual trait; instead, individuals adjust their behaviour according to socioecological conditions. Even if individuals cannot choose their opponents nor assess their fighting ability, our analysis predicts that repeated interactions between individuals with the same fighting ability should lead to a decrease in the level of aggressiveness of individuals with intermediate and strong fighting abilities (that reduce their use of the Hawk tactic, Fig. 2), thereby favouring cooperative interactions.

Phenotypic assortment by body length, a positive correlate of fighting ability (e.g. [33–36]), is a widespread phenomenon notably in shoaling fish species (e.g. [17, 37–39]). Though it may result from an active choice, other passive mechanisms may also drive phenotypic assortment [38]. For instance, individual differences in body size are usually associated with differences in metabolic needs (e.g. [40, 41]) and predation risk [42], and, for that reason, may cause differences in habitat use. Spatial heterogeneity in environmental conditions, therefore, is likely to affect the degree of phenotypic assortment, and consequently, the frequency of cooperative interactions.

Furthermore, when I allowed individuals to modify the structure of the network, individuals with a low and intermediate fighting ability had interest, under most conditions, in breaking connections with stronger opponents, thus forcing contestants with high fighting ability to interact mainly among each other. Yet, because the expected success of individuals depends on their ability to both gain food and escape predators, the number of connections per individual as well as the degree of phenotypic assortment are affected by ecological factors, such as the value of resources and to a lesser extent the risk of predation, but above all by whether individuals can reliably assess the competitive ability of their opponents and adjust their behaviour accordingly. This finding thus demonstrates that behaviour, and particularly the existence of alternative behavioural tactics, is a critical determinant of animal social network structure. Specifically, if individuals may use the Assessor tactic, and hence adjust their behaviour to their chance of winning a fight, individuals with a low fighting ability should systematically break connections with all individuals with a higher competitive ability than their own. In that case, indeed, individuals behave aggressively to get the whole contested resource when they meet an opponent whose fighting ability is lower than their own, preventing individuals with the weakest competitive ability to have any access to food. By contrast, when individuals do not use the Assessor tactic, individuals with a weak fighting ability may benefit from staying connected with stronger competitors if they do not use the Hawk tactic unconditionally, and hence if the cost of fighting is large compared to the value of resources.

If the ability of individuals to assess their relative fighting ability may be constrained by cognitive limitations, ecological factors can also affect the frequency of use of alternative tactics, such as sneaking [43] or dispersing, and hence the network structure. For instance, individuals with a low fighting ability might take advantage of owners who are busy fighting to sneak access of unguarded resources, resulting in an additional cost associated with the hawk tactic. If the use of the alternative Assessor tactic tends to increase the degree of phenotypic assortment, the use of a sneaker tactic, by contrast, should then reduce the level of aggressiveness of strong contestants and thus favours networks with a lower degree of phenotypic assortment. To a lesser extent, I also found that predation pressure could affect the structure of the social network, demonstrating the need not to be limited to one factor (e.g. intraspecific competition) to predict network properties and the resulting ecological and evolutionary consequences. Specifically, the model predicts that increasing predation pressure should slightly increase the total number of connections but with no significant change on the degree of phenotypic assortment. By contrast, observations on different populations of Trinidad guppies (*Poecilia reticulata*) have most often revealed a higher degree of phenotypic assortment under high predation risk [44]. This contradiction may be explained by the fact that I have assumed here that the risk of being detected by a predator is the same for all individuals, regardless of their fighting ability, and does not vary according to their social environment. In other words, the risk of mortality due to predation depends only on the number of neighbours but not on their phenotype. Yet, if odd individuals in a group suffer an increased risk of predation, as appears to be the case in guppies [44], a strong phenotypic assortment could indeed be expected in environments with high predation pressure. These results therefore support the idea that the relative importance of different factors and their respective effect on the social network structure should show strong inter- and intraspecific variability.

## Conclusions

It is increasingly recognized that group social structure has important consequences for ecological and evolutionary processes [45], and, as such, might be a useful tool for conservation [46]. The present study, particularly, suggests that phenotypic assortment by fighting ability would contribute to maintaining individual phenotypic variation. Indeed, given that individuals with a low fighting ability can never win a fight and hence succeed in obtaining food when they meet a stronger contestant, they should be eliminated over time. However, if they decide to interact only with each other, the best strategy becomes the same for all group members, which promotes the coexistence of different phenotypes. Maintaining genetic diversity is critical to allowing wild populations to survive to rapid environmental changes. Findings from the present study thus confirm the importance of taking into consideration the dynamic structure of the social network to predict the frequency of cooperation [47] and the resilience of populations.

### Supplementary Information

Supplementary Material 1.

## Data Availability

The simulation codes of the models have been provided as supplementary material.
