# Supplementary material for: Game theory elucidates how competitive dynamics mediate animal social networks
Source: BMC Ecol Evol. 2024 Aug 30;24:116. doi: 10.1186/s12862-024-02302-6 (PMC11365163; doi:10.1186/s12862-024-02302-6)

**Complementary figures. Game theory elucidates how competitive dynamics mediate animal social networks.**

**Figure S1**. Effects of resource value (i.e. V) on optimal competitive strategy for individuals with a high (A), intermediate (B) and low (C) fighting ability and on the proportion of resources shared without aggression (D). In the figure: $n=6$, $c=2$ and $a=1$. The reported values represent the average frequencies of use of the three tactics estimated from 100 fixed randomly generated social networks for each set of parameter values.


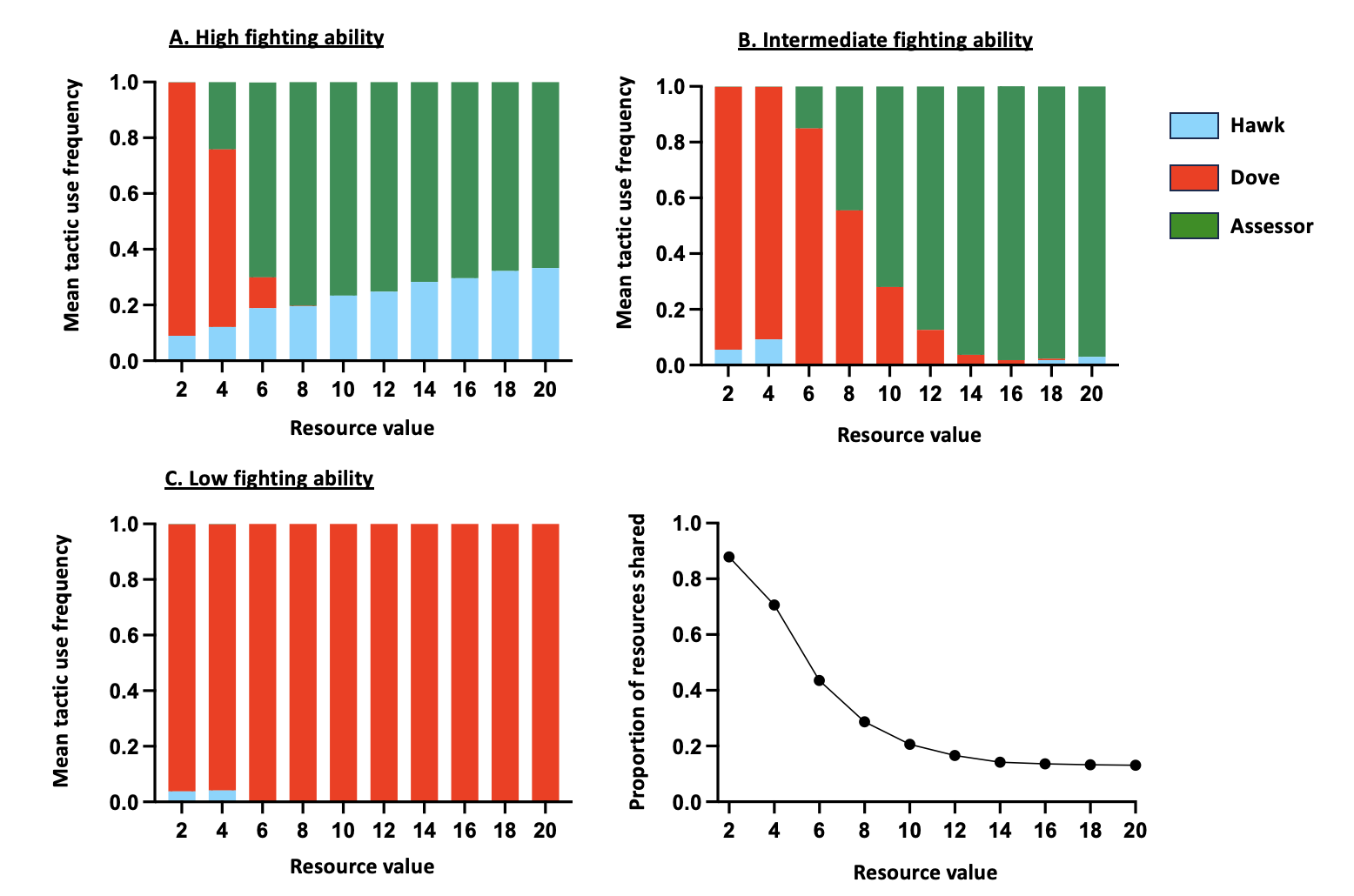


**Figure S2**. Effects of resource value (i.e. V) on optimal competitive strategy for individuals with a high (A), intermediate (B) and low (C) fighting ability and on the proportion of resources shared without aggression (D). In the figure: $n=6$, $c=2$ and $a=10$. The reported values represent the average frequencies of use of the three tactics estimated from 100 fixed randomly generated social networks for each set of parameter values.


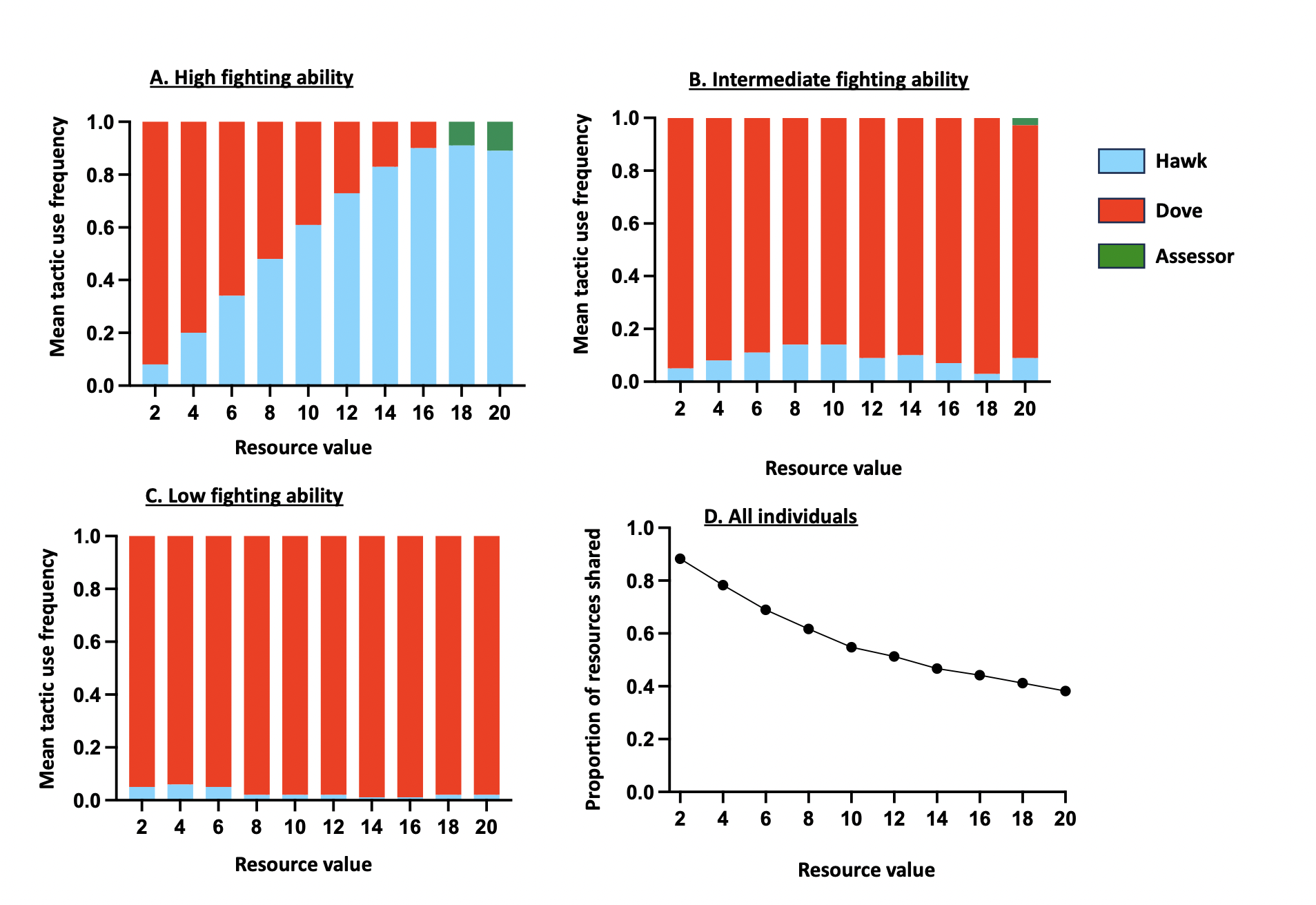

Supplement: Supplementary file 1 — Supplementary Material 1. [file 12862_2024_2302_MOESM1_ESM.docx]
